# A genetic perspective on the recent demographic history of Ireland and Britain

**DOI:** 10.1038/s41431-025-01794-0

**Published:** 2025-02-05

**Authors:** Ashwini Shanmugam, Michael Merrigan, Seamus O’Reilly, Anne M. Molloy, Lawrence Brody, Orla Hardiman, Walter Bodmer, Russell L. McLaughlin, Gianpiero L. Cavalleri, Ross P. Byrne, Edmund H. Gilbert

**Affiliations:** 1 School of Pharmacy and Biomolecular Sciences, Royal College of Surgeons in Ireland, Dublin, Ireland; 2 The SFI Research Ireland Centre for Research Training in Genomics Data Science, School of Mathematics, Statistics and Applied Mathematics, University of Galway, Galway, Ireland; 3 FutureNeuro Research Ireland Centre, Royal College of Surgeons in Ireland, Dublin, Ireland; 4 Genealogical Society of Ireland, Dún Laoghaire, Ireland; 5 School of Medicine, Trinity College Dublin, Dublin 2, Ireland; 6 Genome Technology Branch, National Human Genome Research Institute, National Institutes of Health, Bethesda, MD USA; 7 The Academic Unit of Neurology, School of Medicine, Trinity College Dublin, Dublin 2, Ireland; 8 Weatherall Institute of Molecular Medicine and Department of Oncology, University of Oxford, Oxford, UK; 9 Complex Trait Genomics Laboratory, Smurfit Institute of Genetics, School of Genetics and Microbiology, Trinity College Dublin, Dublin 2, Ireland

**Keywords:** Population genetics, Computational biology and bioinformatics

## Abstract

While subtle yet discrete clusters of genetic identity across Ireland and Britain have been identified, their recent demographic history is unclear. Using genotype data from 6574 individuals with associated regional Irish or British ancestry, we identified genetic communities by applying Leiden community detection. Using haplotype segments segregated by length as proxy for time, we inferred regional Irish and British demographic histories. Using a subset of Irish participants, we provide genealogical context by estimating the enrichment/depletion of surnames within the Irish genetic communities. Through patterns of haplotype sharing, we find evidence of recent population bottlenecks in Orcadian, Manx and Welsh genetic communities. We observed temporal changes in genetic affinities within and between genetic communities in Ireland and Britain. Structure in Ireland is subtler compared to neighbouring British communities, with the Irish groups sharing relatively more short haplotype segments. In addition, we detected varying degrees of genetic isolation in peripheral Irish and British genetic communities across different time periods. Further, we observe a stable migration corridor between north-east Ireland and south-west Scotland while there is a recent migration barrier between south-east and west Ireland. Genealogical analysis of surnames in Ireland reflects history—Anglo-Norman surnames are enriched in the Wexford community while Scottish and Gallowglass surnames were enriched in the Ulster community. Using these new insights into the regional demographic history of Ireland and Britain across different time periods, we hope to understand the driving forces of rare allele frequencies and disease risk association within these populations.

## Introduction

The subtle but distinct fine-scale population structure of Ireland is well-characterised [1–3]. The broad population structure in Ireland segregates along historical provincial boundaries [2, 3]. There is a general east-to-west and a north-to-south gradient of British ancestry with higher proportions observed in northeast and southeast Ireland [3], consistent with demographic and political history of the country [4, 5]. Furthermore, studies have investigated the evidence of migration into Ireland. There is conflicting evidence of Norwegian ancestry in the Irish population, arguing a legacy of Viking activity on the island. Studies using Y-chromosome markers [6, 7] found little evidence of an enrichment of Norse/Scandinavian haplogroups in Irish males with putatively Norse surnames, whilst whole-genome genotypes using modern Scandinavian references show varying evidence of Norwegian ancestry [2, 3, 8]. Recently, ancient DNA shows evidence of Norwegian Viking ancestry in Ireland at the time [9] complicating matters, ancient DNA also suggests significant gene flow from Ireland and the British Isles, into Scandinavia during the Viking age [10].

However, the genetic footprints of demographic history within the island of Ireland, and neighbouring Britain, is relatively under-studied. There is a gap in our understanding of how the effective population size (N_*e*_) and population structure within regions of Ireland has changed over time, and how this compares to neighbouring regions in Britain. This is especially relevant in the era of large biobank samples of the UK population [11], where an understanding of the demographic history of Irish and British communities informs uses of these wider genotype samples, such as expectations around rare variant distribution and burden. Past population sizes affect haplotype diversity and patterns of variation which in turn may contribute to relative risk of complex traits [12, 13]. By understanding population structure and the demographic events that shaped it, we can effectively control for their effects while investigating the dynamics of selection and the association of genotypes to phenotypes within a population [14, 15]. Insights into recent demographic history can also provide vital information about the distribution and burden of rare functional variation, informing expectations. Further, as long-range haplotypes tend to be more recent and can therefore be used to impute rare variant burden in samples to reveal novel genotype-phenotype associations in complex traits and diseases [16–18]. To understand the patterns of genetic variation and the forces that drive it in a population such as Ireland, it is vital to study the effects of demographic events such as migrations [9, 19, 20], to estimate the extent of admixture, and population contractions - e.g. due to famine [20].

In this context, we applied long-range haplotype segment-based methods to study the temporal patterns of: (1) the extent of background relatedness, (2) regional demographic histories, and (3) European ancestry in Ireland and Britain. These insights will provide important information on the temporal footprints of genetic history on the British and Irish genetic landscape and provide a case study of the methods’ use.

## Materials and methods

For a full description of all of the methods and materials, see Supplementary Methods in Supplementary Notes. We briefly describe the methods and materials here. We combined single nucleotide polymorphism (SNP) microarray genotype data from four studies [2, 19, 21, 22] covering 298,420 common SNPs in samples (*n* = 6574) with regional ancestry in Ireland and the United Kingdom. We utilised additional European references [2, 23] to estimate temporal differences in ancestry proportions (194,714 common SNPs and *n* = 13,029). Using haplotypes detected by SHAPEIT4 [24], identity-by-descent (IBD) segments ≥1 cM were detected between haplotypes using refinedIBD [25]. Further, we applied the Leiden community detection algorithm [26] on a network recording total length of IBD ≥ 4 cM between all British and Irish individuals.

We provided a genealogical context to the genetic communities by calculating the relative incidence of surname types based on their origin using a Chi-Squared test. We applied the findings to train a Naive Bayes classifier [27] and validated it using the second-level Leiden genetic community labels. This classifier was then used to predict regional ancestry of Irish participants from the UK Biobank (UKB) [11] dataset (*n* = 7159).

We inferred the demographic histories of genetic communities through the following analyses. We estimated degrees of haplotype sharing within and between genetic communities over time scales by separating IBD and Runs-of-Homozygosity (ROH) segments by length (1–3 cM, 3–5 cM and ≥5 cM). We used IBDNe [28] to estimate changes in N_*e*_ within the genetic communities and recorded IBD-sharing between every British and Irish group, and regional European clusters then applied PCA biplots to decompose these contributions over the three IBD bins described previously.

## Results

### Population structure in Ireland and Britain

Using the largest collection of Irish reference haplotypes with geographic provenance assembled to date (*n* = 3502), combining datasets of Irish and British ancestries (Table 1), we identified a total of 25 genetic communities using the Leiden network community detection algorithm [26] over three levels of recursive clustering (see Methods).

**Table 1:** Summary of datasets.

Dataset	Population	Geographic data	
		Present	Absent
ALS case control cohort	Northern Ireland	7	0
	Republic Of Ireland	533	445
Irish DNA ATLAS	Northern Ireland	24	0
	Republic Of Ireland	166	5
People of the British Isles	England	2405	29
	Isle of Man (IoM)	56	0
	Northern Ireland	69	0
	Orkney	136	1
	Republic Of Ireland	6	1
	Scotland	152	30
	Wales	290	5
TRINITY student study	Republic of Ireland	0	2214

**Fig. 1:**
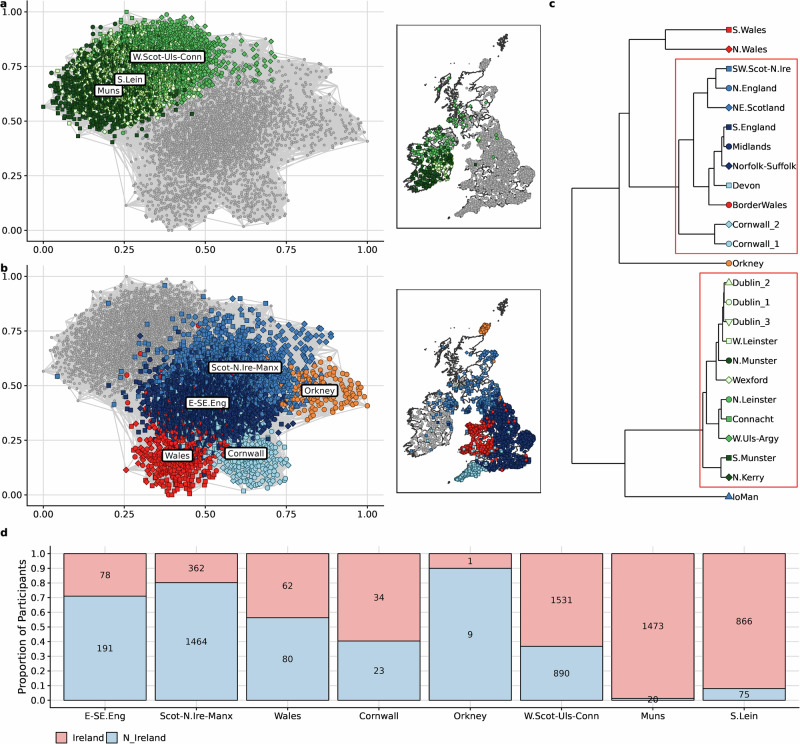
Genetic structure in Ireland and Britain. **a**, **b** A graphical projection of the network of genetic communities detected in Ireland and Britain. Every point on the plot is an individual with British or Irish ancestry and the colours of the points indicate genetic community membership. Each line, or edge, is the average length of IBD segments shared between a pair of individuals with line thickness corresponding to the total length of segments shared. For each individual, we show the top 10 edges for plotting. The maps adjacent to the networks show the geographic origin of individuals (see Methods for more details). **c** hclust dendrogram of 3rd level genetic communities showing genetic similarity. The red rectangles around the branches indicate high certainty grouping and the colour of the points indicates the 2nd level genetic community and shape of the points indicate the 3rd level genetic community. **d** Barplot depicting the predicted regional ancestry of Irish and N.Irish participants from the UK Biobank. The *x*-axis shows the second-level genetic communities within Ireland and Britain to which samples were assigned by our classifier. The colours in the stacked barplot indicate the reported birthplace of the UK Biobank participants and the *y*-axis displays the proportion of the samples assigned to the regional ancestry group from each birthplace.

Confirming previous reports [2, 3], these genetic communities segregated by geography, and were thus assigned labels to broadly reflect these geographic affinities (Supplementary Table 1). The first recursion classified individuals into two communities (Supplementary Fig. 1a), each with predominantly Irish and British membership respectively. The second recursion largely split the communities by historical or administrative boundaries—provinces within Ireland (*n* = 3) and generally countries within the UK (*n* = 5) (Fig. 1a, b). The third recursion identified fine-scale structure within these groups (Supplementary Fig. 1b–i). We identified previously unobserved genetic structure within Ireland (N.Kerry, S.Leinster, three Dublin communities).

We performed hierarchical clustering using *pvclust* [29] to assess the reproducibility of this genetic structure. From this, we find supporting evidence that these communities represent meaningful divisions in our data (Fig. 1c and see Supplementary Note 1 for full analysis). Further permutations of total-variation distances [19] also support the robustness of the communities (*p*-value << 0.01, Supplementary Table 2).

We demonstrate the value of these findings in the context of the UK Biobank [11]. Using UKB individuals with an Irish or Northern Irish birthplace and Irish ancestry (“UKB Irish”) (see Supplementary Methods, “Datasets”), we show that the self-reported “white British” or “white Irish” ethnicity label poorly captures the diversity of Irish ancestry (Fig. 1d). We trained a Naive Bayes classifier to predict regional Irish and British ancestry of the UKB Irish, dividing our dataset into training and validation subsets (see Methods). We find that 67.81% of these Irish or Northern Irish-labelled UKB participants are predicted to have regional ancestry most commonly found in the Republic of Ireland. Using our second-level clusters (Fig. 1), we predicted 33.82% of UKB Irish to have Western/North-Western Irish ancestry, 20.85% Southern Irish ancestry and 13.14% Eastern Irish ancestry. Of the remaining 32.19%, 6.68% were classified into one of many British-majority ancestry clusters, and 25.51% were classified in the Northern Irish/South-West Scottish/Manx-like group (Supplementary Table 3). This group contained communities of shared Northern Irish and Northern British ancestry. This northern Irish-ancestry UKB group was predominantly made up of those UKB Irish born in Northern Ireland (80.17% vs 19.83% who were born in the Republic of Ireland).

#### Genealogy and population structure

Genealogical data can also provide additional context to genetic communities detected—the Irish DNA Atlas allowed us to test the preponderance of different surname origins (i.e. English, Scottish, Irish) within each of the Irish third-level clusters (*p*-value < 2.2e–16 and adjusted Cramer’s V of 0.20 indicating a strong association) (Supplementary Fig. 3a). We observed a significant enrichment of Scottish and Gallowglass surnames in the SW.Scotland-N.Ireland community—matching historical settlement of Scottish mercenaries in that area between the 13th and 16th centuries [4]. Within the N.Munster and N.Kerry communities, we observe an enrichment of Welsh surnames and in Wexford, we find an increase of English, Anglo-Norman and Scandinavian surnames which possibly may reflect older Anglo-Norman settlements in these regions [5]. We observe a slight preponderance of Scandinavian surnames in the southeastern Irish communities. When we extended this analysis to individual surnames (Supplementary Fig. 3b), we observed an enrichment of “Walshes” in N.Kerry and W.Leinster, “Sullivans” and their variants in N.Kerry and S.Munster, “Ryans” in N.Munster, “O’Donnells” in W.Ulster-Argyll.

### Demographic profiling of genetic communities across Ireland and Britain

Using the regional genetic communities detected, we sought to comparatively profile the demographic histories of these communities across different timescales by using IBD segment length bins as proxy for time. We further used these bins to estimate temporal migration rates in Ireland and the UK, and the ancestral contributions from other European populations over three time periods in recent history from 100, 45, and 15 generations ago. These were estimated to be ~3000 years, 1200 years and 450 years ago respectively which coincide with the late Bronze to early Iron age in Ireland, the presence of Norse-Vikings and Anglo-Normans in Ireland, and reformation and Plantations in Ireland respectively. Since the time frames of IBD segment bins have wide ranges [30, 31], we refer to the bins by their length noting their age relative to each other, i.e. more recent, older etc. We complemented these methods with estimating levels of ROH for signals of inbreeding and/or isolation (see Methods). Lastly, we estimated changes in the effective population (N_e_) sizes in recent history using IBD segment data (See Supplementary Note 2).

#### Changes in structure across time

**Fig. 2:**
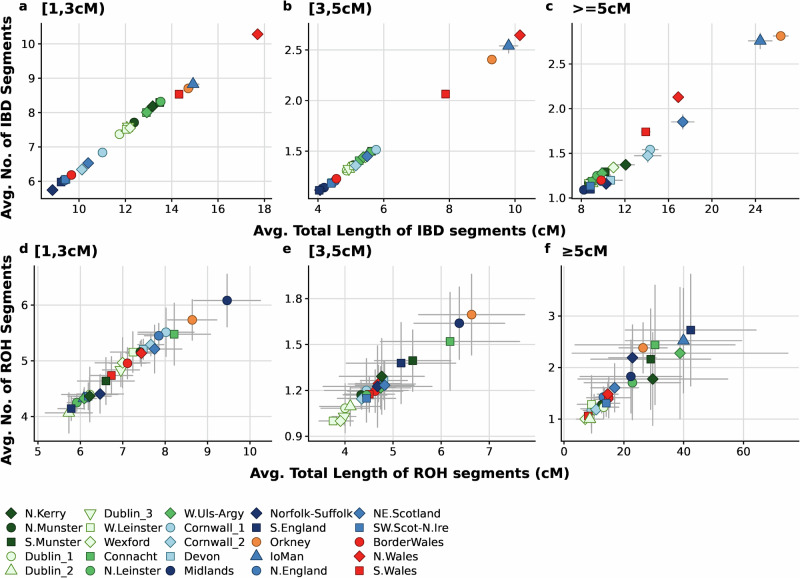
Inferring demographic history from the extent of haplotype and ROH sharing. **a**–**c** The scatter plots show the extent of IBD shared within every 3rd level genetic community after the IBD segments are segregated based on their length. Each point represents a 3rd level genetic community. The *x*-axis shows the average total length of IBD segments shared between pairs of individuals within the genetic community while the *y*-axis indicates the average number of IBD segments shared between pairs of individuals. **d**–**f** The scatter plots show the extent of ROH shared within every 3rd level genetic community after segregation based on their length. We plotted the average number of ROH segments shared between pairs of individuals in the community and the average total length of ROH segments shared between pairs of individuals to identify population isolates. The grey lines projecting from each point indicate the 95% confidence interval.

Comparing IBD sharing profiles in Ireland and Britain, we find evidence of shifting demographic relationships over time. We observe that ~100 generations ago (IBD length bin [1,3 cM)) Irish communities on average shared more and longer IBD segments than most English (right-tailed *t*-test *p*-value < 2.2 × 10^−16^) and Scottish (right-tailed *t*-test *p*-value < 2.2 × 10^−16^) communities. (Fig. 2a and Supplementary Table 4). Further, sharing patterns within Irish communities is subtler compared to their British counterparts, suggesting greater homogeneity (Fig. 2a–c and Supplementary Table 4), and demonstrating subtle shifts in population structure. We observed that elevated shared IBD levels in Ireland (Supplementary Fig. 4a–c and Supplementary Fig. 5a–c) is primarily due to sharing within the [1,3 cM) bin which decreases as the IBD bin lengths increase. This indicates an older signal of relative isolation in the Irish communities compared to their general British counterparts. This corroborates our *F*_ST_ findings where the Irish communities have a lower median *F*_ST_ of 2.20 × 10^−4^ compared to UK communities (*F*_st_ = 9.51 × 10^−4^ ; Supplementary Note 1 and Supplementary Table 5).

In the [1,3 cM) IBD length bin, we observed that IBD sharing increases as we move from the east to the west of Ireland (Fig. 2a), signalling greater isolation in the west ~100 generations ago which could be explained by smaller population sizes in the region. Within-community sharing in Ireland however, remains significantly lower than highly isolated communities such as Orkney, N. and S. Wales and the Isle of Man (IoM) across all length bins (Fig. 2a–c) (left-sided *t*-test *p*-value < 2.2 × 10^−16^). Irish communities have a similarly high degree of haplotype sharing as the Scottish, Manx, Welsh, and Orcadian groups in the [1,3 cM) IBD bin (Supplementary Figs. 4a & 5a). However, while the Irish show equivalent values with the Manx and SW. Scottish groups in the [3,5 cM), it diminishes with the NE.Scottish and Orcadian genetic communities in IBD bins ≥3 cM. In contrast, the N.Welsh genetic community appears to share slightly more IBD segments in the more recent [3,5 cM) and ≥5 cM bins (Supplementary Figs. 4a–c & 5a–c & 6a–c), as do the Cornish communities. These changing affinities between populations across time likely reflect complex demographic relationships which may be better understood by considering migration rates and isolation across time.

Population size and isolation can leave detectable signals on the distribution of ROH segments in a community. ROH segments in the [1,3 cM) length bin indicate signals of isolation in the English Midlands, Orkney, Cornwall, and Connacht genetic communities 100 generations ago (Fig. 2d). This signal persists in the [3,5 cM) ROH length bin for the Midlands, Orkney and Connacht genetic communities. However, we observed a recent inflation of parental relatedness in the S.English, Manx, and W.Ulster-Argyll groups in >5 cM length bin. Overall, ROH shared within the Irish, Scottish and English groups are comparable (Supplementary Table 6), whereas specifically the Orcadian, Manx and Welsh groups share higher levels of ROH. Complementing this, the lower SNP-based inbreeding coefficients (F_IS_) compared to inflated ROH-based coefficients (F_ROH_) for the populations indicate that the observed patterns of haplotype-sharing is likely due to small N_*e*_ than consanguinity [32] (Supplementary Fig. 8).

#### Changes in population size, isolation and migration

**Fig. 3:**
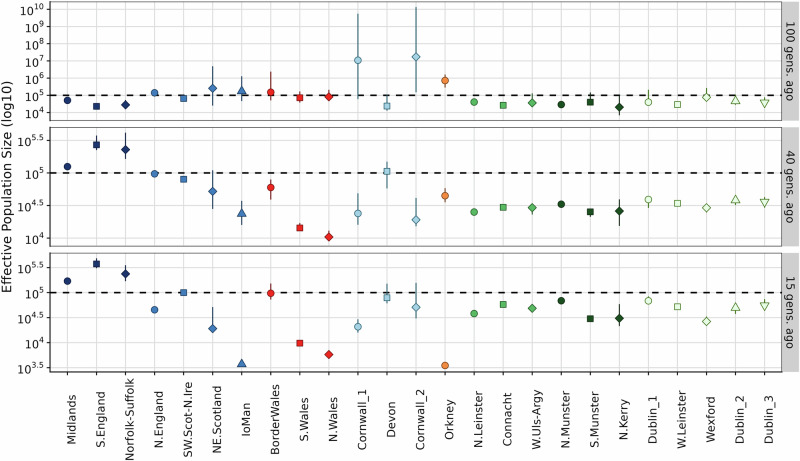
Effective population size (N_*e*_) estimates of communities in Ireland and Britain. The dot plots show snapshots of effective population size estimates of the genetic communities in Ireland (shades of green) and Britain (shades of blues, red, and orange) corresponding to the IBD length bins [1,3 cM), [3,5 cM) and ≥5 cM. The third-level genetic communities in Ireland and Britain are represented along the *x*-axis and are indicated by the shape and colour of each point. The *y*-axis represents the effective population size estimates (N_*e*_) of the third-level genetic communities and is in log_10_ scale. The coloured lines from each point represent the 95% confidence intervals of each N_*e*_ estimate.

Haplotype-sharing patterns within Ireland and Britain can provide insights into the sizes and movements of populations, providing context for observed genetic structure. Irish communities show relatively high IBD-sharing with little variation in sharing between Irish communities (Supplementary Fig. 4) indicative of low N_*e*_ (Fig. 3). Using IBDNe, we estimate N_*e*_ over time. We observed that this relatively homogenous sharing pattern is reflected in the similar N_*e*_ estimates of the Irish communities across the island 100 generations ago (Supplementary Fig. 7a–c, Fig. 3). Two-thirds of the Irish communities show a reduction in N_*e*_ 40 generations ago, specifically in the south and east of Ireland. The Wexford, N. Leinster, and S. Munster genetic communities which show 38–72% reduction in population size between 100 and 40 generations ago (Supplementary Table 7). We see a further reduction of 10% in the effective population size in the Wexford genetic community while there is an exponential increase (18–100%) in N_*e*_ in the other Irish genetic communities (Supplementary Table 7). In addition, the N.Kerry community demonstrates a population expansion and followed by contraction within the past 30 generations (Supplementary Fig. 6a) which could be due to its small membership or may reflect cryptic relatedness just above our threshold of relatedness filtering (see Supplementary Methods).

Within the UK, there are elevated levels of haplotype sharing across varying bins of IBD or ROH length. The Orcadian, Manx, and the N. and S. Welsh genetic communities (and to a lesser extent Cornwall) demonstrate features of isolation (Fig. 2a–c and Supplementary Table 4). This is further reflected in their low N_*e*_ over time, which are consistently lower than other British communities and show evidence of recent population contraction (Fig. 3, Supplementary Fig. 7d–g and Supplementary Table S7), especially N.Wales. By performing a PCA on an IBD-sharing matrix in length bins [1,3 cM) and [3,5 cM), principal components 1 and 2 resolved these communities from the Irish and other British communities (Supplementary Fig. 6a–c). PCA on the average total length of IBD shared also shows that the Scottish, Manx and N.Irish groups are on a west-to-east cline between the Irish communities and British communities. The Cornish communities appear to be more recently isolated (Supplementary Fig. 6d) with their population sizes exponentially decreasing over the course of 80 generations, matching an enrichment of within-community IBD sharing >5 cM. These Cornish communities also share marginally more IBD with the Devon community and then the S.English community when compared to the other British communities (Supplementary Figs. 4 & 5). In contrast, the S.English communities see a steady increase in N_*e*_ over time (Supplementary Fig. 7e), though with a common contraction in N_e_ followed by a recovery around 10 generations ago that is observed in nearly all clusters in Britain and Ireland.

**Fig. 4:**
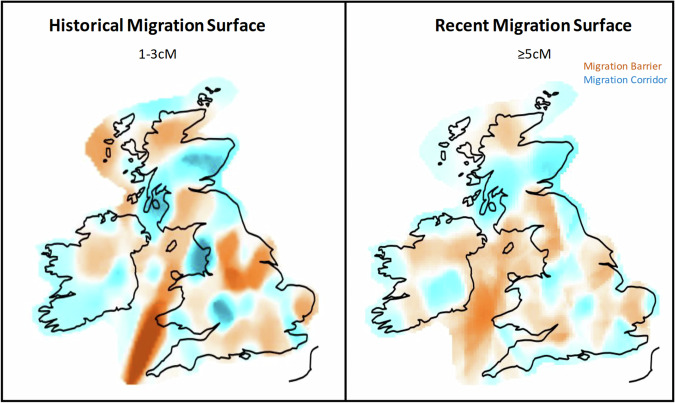
Temporal migration rate surfaces in Ireland and Britain. The plots show the estimated migration rate surfaces in Ireland and Britain in two time periods. The barriers are coloured in red while the migration corridors are coloured in blue. The outlines of the countries were sourced from Global Administrative Areas [35]. The figure was produced using the plot_maps function in the plotmaps package in R (ver. 4.2.1).

The observations of isolation in periphery communities above are reinforced by migration rate surfaces estimated from MAPS [30] (Fig. 4). We observed that the Orkney islands, IoM, Wales, Cornwall and Devon show low migration rates to and from the mainland both in the older [1,3 cM) and more recent (≥5 cM) IBD bins, further supporting their isolation. The N_*e*_ dips consistently over time in NE. Scotland, IoM, Orkney and Cornwall (Supplementary Fig. 6). Stable migrational barriers between north and south Wales, confirming long standing structure within Wales. Additionally, we detected little migration between the Scottish Lowlands and Highlands and Britain in the older [1,3 cM) IBD bin. However, we observed a new migration corridor opening between the Scottish Lowlands and N.England in the more recent IBD bin (≥5 cM), while the Highlands remain isolated from the rest of Britain (Fig. 4a, b). Interestingly, and supportive of our IBD sharing results, N.Wales exhibits a historically smaller N_e_ around generations 30–50 than Orkney or the Isle of Man.

To further contextualise the structure and isolation in Ireland, migration rate surfaces (Fig. 4) show a consistent, stable migration corridor between northeast Ireland and southwest Scotland, demonstrating the isolation of the island and matching previous genetic evidence of migration to and from these regions [2, 8]. This genetic data also supports Scottish-origin surname distributions in the Irish DNA Atlas (Supplementary Fig. 3). The within-Ireland migrational cold spots however, shifted across time from central-West Ireland in the [1–3 cM) IBD bin to isolating Leinster from the rest of the island 15 generations ago (Fig. 4), separating Kerry from Galway, which may explain isolation in Connacht. Additionally, in the north of the country, a shift in migration corridors links the west and east of Ulster, likely reflecting gene flow from Scotland across Ulster.

#### Continental European ancestry in Irish and British communities

We also investigated the European ancestry contributions to our genetic communities across time using PCA of European IBD sharing (Supplementary Fig. 9 and Supplementary Table S8) to complement the previous results [2, 3, 19]. In the oldest [1,3 cM) IBD bin, we observe that English genetic groups separate out from Irish communities on axes driven by Germanic-Swedish-Norwegian ancestries. Scottish-N.Irish communities demonstrate more Swedish influence while there appears to be strong north and northwestern Norwegian influence in the Manx and Orcadian groups (Supplementary Fig. 9a and Supplementary Fig. 10–13a–c). In more recent history (>5 cM) however, there appears to be more of a west-Germany ancestry signal in the English communities while there is more of a Swedish-Finnish-Norwegian component in the Scottish groups. The Norwegian signal persists in the Manx and Orcadian groups (Supplementary Fig. 9b and Supplementary Fig. 10–13a–c).

Focussing on the contributions of European ancestry to Ireland over time, we find a strong signal from north Norway and western France and to a lesser extent from Sweden in the Irish communities in the [1–3 cM) IBD bin (Supplementary Figs. 10–13). There appears to be a more recent contribution of ancestry in the [3,5 cM) IBD bin from North-North-West Norway and Sweden in Ireland, specifically in the W. Leinster community (Supplementary Figs. 10–13). This can be seen as further confirming recent reports of gene flow from Ireland to Norway [9].

## Discussion

In our study, we used patterns of IBD-shared between 6574 individuals with regional ancestry to infer recent demographic histories across Ireland and Britain—providing context for genetic communities described here and in previous literature [1–3]. We leverage IBD segments to confirm that genetic structure and regional genetic differences in Ireland are subtler compared to Britain, and then show temporal changes in regional migration rates and population sizes in Ireland and Britain. While IBD sharing is possibly lower resolution than haplotype painting, it is more exact since matches are only seen if there is a shared ancestor, and the length distributions are more easily mapped to time periods [30, 31]. We detected signals of population isolation in peripheral genetic communities (e.g. Wales, IoM and Orkney) and growth in others (e.g. South England). Additionally, we provide genealogical context to the findings of these Irish genetic communities through analysis of surnames.

In agreement with previous studies [1–3], we found the genetic communities detected largely divided into Irish and British-like genotypes with sub-communities that resolved along provincial/historical kingdom boundaries in Ireland and country/administrative boundaries in the UK. With geographic provenance from our dataset, we predicted regional Irish or British ancestry within larger datasets such as the UKB, demonstrating that well-characterised reference datasets provide greater value while studying regional health genomics in biobanks. Our findings show that the UKB self-reported “white British” or “white Irish” group is biassed, being weighted towards the north of the island which accounts for nearly 60% of the predicted Irish-like participants. While the UKB is a large repository of Irish ancestry, any fine-scale understanding of regional ancestry of UKB Irish samples is possible with a well-annotated reference dataset.

Based on the genetic structure of Ireland and Britain, we leveraged haplotype sharing to characterise time-sensitive changes in the demographic history of Ireland and Britain. Modest yet consistently elevated levels of IBD shared between Irish regions and their correspondingly smaller N_*e*_ demonstrates the relative isolation of Irish groups compared to England, which suggests a common demographic history across the island. Further, genealogical data from the Irish DNA Atlas highlights the regionality of several surnames in Ireland, thus tying genealogical footprints of people to genetic clusters.

We also observed features of isolation in British regions such as IoM which shows a signal of isolation similar to Orkney, although smaller in magnitude. Interestingly, we observed elevated levels of IBD sharing between the Irish and Scottish, Welsh, Manx and Orcadian groups in the smaller IBD bins that disappeared in the ≥5 cM IBD bin, suggesting a shared historical Gaelic (or Norse-Viking signal) in these populations. The affinity of the Manx group in our dataset to the Irish population is in direct contrast to the report from Gilbert et al. in 2019 which used different Manx samples and showed a high proportion of English ancestry in Manx individuals. The observed discrepancies between our observation and that reported by Gilbert et al. could be due to different ascertainment schemes from IoM, sampling two communities with differing affinities to England/Ireland.

Additionally, our time-sensitive IBD-bin method is able to highlight novel results in Wales. We found an excess of within-group sharing in smaller IBD-length bins which disappears in more recent bins, suggesting the primary source of genetic isolation observed in Wales is older than in Orkney or IoM, who have experienced more recent isolation. This Welsh isolation is also stronger in north Wales, which is considerably separated from the south. By inferring regional demographic histories, we confirmed the Orcadian genetic community has historically been genetically isolated for the past 100 generations. We also observed a consistent gene flow between the Orcadian community and the northeast Scottish group.

By estimating contributions of European ancestries in the Irish and British genetic communities across timescales, we confirm evidence of gene flow between Scandinavia and the English, Scottish and Irish groups, refining this observation to the smaller IBD bins. This contrasts with the Orcadian and Manx communities where the signal is observed across all IBD bins. We also detected an affinity between modern populations in northwest Germany and the English communities in the smaller IBD bins, consistent with the influence of Germanic migrants in the Migration Period [5] in the region [19]. Further, by separating out signals of sharing with European groups, extending previous findings using haplotype “painting” of chromosomes, we shed light on the changing landscape of European affinity across the islands. Additionally, we confirm results from previous papers which have demonstrated genetic links between western France and Ireland [2, 3, 33, 34]. Lastly, our results are relevant to the question of the genetic legacy of Norse Viking activity on the modern Irish genetic landscape. We observed a higher similarity between Irish genetic communities and modern Norwegian haplotypes in smaller IBD segment bins that is concurrent to the signal in Orkney and the Isle of Man and consistent with a Norse-Viking origin. Indeed, analysis of surnames within the Irish DNA Atlas shows a minor inflation of putatively “Scandinavian” surnames in the southeast of Ireland where a number of Viking settlements were founded - though this should be treated with caution as putative origins of surnames are sometimes unclear and adoption of surnames may further obfuscate any link between surname and gene flow. Recent work with ancient DNA in Scandinavia has demonstrated significant gene flow from Ireland and the British Isles into Viking-era Scandinavia [9, 10] including the regions of Norway previously shown to have an excess sharing in Ireland [3, 8]. Overall, our results here are consistent with the current model that an inflation of present-day Norwegian sharing in present day Ireland is primarily due to this historical Irish gene flow to Viking-era Norway.

While interpreting the results, we considered a relatively simple approach of using IBD segment length as a proxy for age and IBD shared as a representation of population size [28, 30]. There could be other, more complex demographic scenarios that explain our results. In addition, IBD segments of length 1 to 3 cM have a higher proportion of noise to signal—our inferences of demographic history a 100 generations ago might not be an accurate representation of that period. The uneven sampling of participants across Ireland and the UK has led to overrepresentation within some of the identified genetic communities. Samples from Scotland were predominantly from the north-east and south-west of the country, preventing analysis of known and finer-scale Scottish genetic clusters as demonstrated by Gilbert et al. [8]. Generally, the British sampling was more rural which limited analysis of large urban areas. Nearly a half of our samples were of Irish genealogical origin, and the majority of samples after that were of English origin, resulting in greater resolution in community detection in those places compared to Scotland. Further, we had few samples from north-west and south-west of Ireland, although this would match population densities across the island.

In conclusion, we show that the regional genetic differences in Ireland are subtle and can largely be attributed to older genetic sharing whereas the observed genetic differences in Britain are largely along administrative boundaries. Through haplotype-sharing analysis, we identified genetically isolated regional Irish and British communities over different time periods, and highlighted the signals of contracted population size in Wales. Therefore, our demographic history results and the changing affinities of regional Irish and British populations over time motivate the study of rare variants within them, to determine if they indeed stratify by patterns that match the demographic history of the population. Additionally, we demonstrated that these results can be used to predict regional ancestry of Irish participants in larger datasets such as the UK Biobank.

## Supplementary information

Supplemental Notes

Supplemetal Figures

Supplemental Table 1

Supplemental Table 2

Supplemental Table 3

Supplemental Table 4

Supplemental Table 5

Supplemental Table 6

Supplemental Table 7

Supplemental Table 8

## Data Availability

Please contact the corresponding author, Dr Edmund Gilbert, to access the Irish DNA Atlas genotype data. For the other datasets used in this study, please see the published articles for details; Trinity Student Study dataset [21], the Irish ALS Case Control Cohort [3], the Peoples of the British Isles dataset [19], and the WTCCC2 Multiple Sclerosis dataset [23]. The UK Biobank can be accessed by applying through the UK Biobank portal.
