# Supplementary material for: A genetic perspective on the recent demographic history of Ireland and Britain
Source: Eur J Hum Genet. 2025 Feb 5;33(4):538–45. doi: 10.1038/s41431-025-01794-0 (PMC11986122; doi:10.1038/s41431-025-01794-0)
Supplement: Supplementary file 1 — Supplemental Notes [file 41431_2025_1794_MOESM1_ESM.docx]

TITLE: A genetic perspective on the recent demographic history of Ireland and Britain

# **SUPPLEMENTAL NOTES**

## Supplemental Methods

### Datasets

Genotype data from individuals of British and Irish ancestry was accessed from four studies: the Irish DNA Atlas (*n* = 198) (Atlas) [(1)](https://www.zotero.org/google-docs/?p9nUE2), the Trinity Student Study (*n* = 2,232) (TSS) [(2)](https://www.zotero.org/google-docs/?7mgL86), the Trinity Irish ALS case-control cohort (*n* = 991) (ALSCCC) [(3)](https://www.zotero.org/google-docs/?xeAHlj) and an updated version of the People of the British Isles dataset (PoBI) [(4)](https://www.zotero.org/google-docs/?pLdIuG) with additional genotypes (*n* = 6,294). In addition, we accessed European reference haplotypes (*n* = 6,916) from the WTCCC2 Multiple Sclerosis dataset (WTCCC2-MS) to estimate temporal differences in ancestry proportions.

Geographical provenance was available for two datasets: the Irish DNA Atlas and the PoBI datasets. The great-grandparents of all individuals recruited for the Irish DNA Atlas were born within 50 km of each other [(1)](https://www.zotero.org/google-docs/?IDsFz5). As for the PoBI dataset, the grandparents of all individuals in the study were born within 80 km of each other [(4)](https://www.zotero.org/google-docs/?wpsypY). We assigned geographic provenance to the participants with the average coordinates of birthplaces of the great-grandparents or each participant for Irish DNA Atlas and those of the grandparents for PoBI. The geographic data for the place of residence was available for approximately half of the individuals in the ALSCCC dataset.

The UKB Irish subset were identified from the UKB from the following criteria. Individuals were selected based on; 1) a place of birth in Ireland or Northern Ireland (UK) (UKB field 1647, values 4 or 5), 2) self-reported ethnic background of “white”, “white British”, “white Irish”, or “white other” (UKB field 21000, values 1, 1001, 1002, or 1003). The first eight genetic principal component coordinates of these individuals (using UKB field 22009) were used in a dbscan[(6)](https://www.zotero.org/google-docs/?NcQ3GJ) to identify a core, dense, cluster corresponding to a common Irish genetic ancestry. Outliers were removed, leaving the “UKB Irish subset”.

We obtained the necessary permissions to work with the datasets used in our study. Please refer to the original papers for the ethics statements [(1–5)](https://www.zotero.org/google-docs/?1pJd4l).

### Quality Control

Using PLINK (ver. 1.9) [(7)](https://www.zotero.org/google-docs/?EgIlLy), we first processed individual datasets by excluding any strand-ambiguous SNPs and included autosomal SNPs. For the Irish-British analysis, we combined the Atlas, PoBI, TSS, and ALSCCC datasets. For the European ancestry analysis, we combined the Atlas, PoBI, TSS, and ALSCCC datasets with the WTCCC2-MS dataset.

Datasets were merged using the PLINK command --merge. We removed SNPs with minor allele frequency (MAF) <2%, missingness >5%, and a Hardy-Weinberg Equilibrium (HWE) p-value <1e-6. We excluded individuals with SNP missingness >5% and excluded SNPs in regions known to be in linkage disequilibrium [(8)](https://www.zotero.org/google-docs/?qNIy5x). Pairs of related individuals (up to the 3rd degree) were identified using KING (ver. 2.2) [(8)](https://www.zotero.org/google-docs/?evAXk2) and one random individual from each pair was removed. To calculate an unlinked PCA, we utilised a set of SNPs that were pruned with respect to linkage disequilibrium using the PLINK command --indep-pairwise 1000 50 0.2, performing PCA using the --pca PLINK command on samples for the Irish-British and the European ancestry analyses.

The final Irish-British dataset had 6,574 samples and 298,297 markers while the European dataset had 13,029 samples and 194,714 markers. This level of coverage has been found to be sufficient when considering longer (i.e., > 3cM) segments of IBD or ROH [(9)](https://www.zotero.org/google-docs/?I5T5fQ). The geographic data for the Trinity ALS dataset has been jittered to preserve anonymity. The combined Irish-British dataset and the Irish subset from UK Biobank had 12,690 samples over 69,788 markers.

### Phasing and IBD Segment Detection

Unphased genotypes were converted to VCF files using PLINK and separated by autosome. Using SHAPEIT4 [(10)](https://www.zotero.org/google-docs/?I9LrN3), we phased the samples with default settings and the GRCh37 build genetic map for recombination distances generated from HapMap. IBD segments ≥1cM in length were detected using refinedIBD [(11)](https://www.zotero.org/google-docs/?R7tSfL) with the default settings and adjacent IBD fragments within 50kbp and 0.06cM of each other were merged using the merge-ibd Java script — merging putative IBD segments which were broken by either phase or genotyping errors. To extract data reflective of different time periods, autosomal IBD data was merged and subsets were created based on length of IBD segments. The extent of IBD shared between pairs of individuals was estimated by calculating the average total length of IBD shared and the total number of IBD segments [(12)](https://www.zotero.org/google-docs/?vfpg9d).

### Population Structure Detection

Using the summarised IBD data , we first constructed a network graph using every individual as a node and the average total autosomal IBD length shared between a pair of individuals as the edge (*igraph* package in R [(13)](https://www.zotero.org/google-docs/?8ckQnD)). We exclude outlier connections of oversharing to account for cryptic relatedness, excluding connections with a sum of all segments ≤ 99.995 percentile of IBD length - 122.87 Mbp. The Leiden community detection algorithm [(14)](https://www.zotero.org/google-docs/?ejHJpJ) was then applied on this network. We used the *rleiden.community* function within the *leidenAlg* package in R [(15)](https://www.zotero.org/google-docs/?2krE4s) with a max.depth of 3 (i.e. 3 recursions of the clustering process) and a *min.community.size* of 100 (i.e. communities of 100 individuals were not divided in a subsequent recursion). The communities were labelled reflective of their geographic membership.

To estimate reproducibility of these Leiden clusters, we ran the leiden.community function with the same settings for 100 iterations. The Leiden algorithm begins with each individual node forming their own cluster, and the first merging step being random. Performing replicates of the Leiden algorithm allows us to test the reproducibility of the original clustering pattern. Using the genetic communities from the first iteration as the basis, we calculated the proportion of individuals who were grouped together in the same genetic community at the three levels of recursion for every subsequent iteration, and then averaged across the original clusters.

To investigate the hierarchical structure of the genetic communities, we computed Euclidean distances between the groups using the haplotype-sharing matrix generated by pbwt-paint [(16)](https://www.zotero.org/google-docs/?pAMH03) between pairs of individuals in the dataset to generate a distance matrix using the dist function in R. The *hclust* function was applied on this distance matrix to generate the final dendrogram. We then validated the dendrogram using the pvclust function from the pvclust package with 10,000 bootstrap resampling. We estimated genetic distances between communities using F_ST_ as calculated in the admixtools package in R [(17)](https://www.zotero.org/google-docs/?IkdpbD), and measured cluster robustness with TVD estimates calculated as described in the PoBI study [(4,18)](https://www.zotero.org/google-docs/?L76pkI).

### Predicting Regional Ancestry within the UK Biobank

We used PLINK to perform principal component analysis after combining our Irish and British genotypes with the UKB Irish subset. We then randomly split the Irish-British reference data 7:3 to form training and validation datasets. We scaled the first five principal components of the training set and trained a Naive Bayes classifier with the data and their corresponding second-level genetic community label with the default parameters (*naiveBayes* function in the *e1071* [(19)](https://www.zotero.org/google-docs/?OSlGbT) R package). To evaluate the accuracy of the model, we predicted the second-level genetic community label of the validation dataset and calculated the concordance between the observed and the predicted label. The median accuracy of this model was 66.9%. We predicted the second-level genetic community labels for the Irish subset of UKB using the model.

### Estimating Extent of Genetic Relatedness

For every genetic community, we first created subsets of IBD segment sharing data based on the length of IBD segments: IBD segments of (a) between 1 to 3cM ([1,3cM)), (b) between 3 to 5cM ([3,5cM)) and (c) greater or equal to 5cM (5<=IBD). These approximately correspond to (a) 100 generations ago, (b) 40 generations ago, and (c) 15 generations ago respectively [(20)](https://www.zotero.org/google-docs/?ZG0GSj). To characterise their histories, we then plotted the average number of IBD segments versus the average length of total IBD segments shared for the different IBD length bin sizes. We calculated the total length and number of IBD segments shared between pairs of individuals in the dataset. Then, we generated summary statistics for every pair of 3rd level genetic communities. We generated a heatmap each after calculating the z-scores of the average total length and number of IBD segments respectively. Additionally, we generated principal components using the average total length of IBD segments using the *prcomp* function in R.

To estimate consanguinity in the Irish and British genetic communities, we estimated ROH within every genetic community using --homozyg with a minimum SNP count of 50, a scanning SNP widow size of 50 SNPs, minimum length of 1500 kb, maximum internal gap of 1000 kb length, a maximum inverse density of 50kb/SNP, a maximum missing calls in scanning window hit of 5, and a maximum number of heterozygous hits in scanning window of 1. We additionally estimated the F_ST_ [(21)](https://www.zotero.org/google-docs/?vutPYD) statistic, and the F_ROH_ [(22)](https://www.zotero.org/google-docs/?cEAPiL) and F_IS_ [(21)](https://www.zotero.org/google-docs/?emrPK6) statistics for every 2nd level and 3rd level genetic community.

### Estimating Effective Population Sizes

To estimate changes in effective population sizes over time, we used IBDNe [(23)](https://www.zotero.org/google-docs/?WPM1a1). We selected IBD segments ≥4cM shared between pairs of individuals within the 3^rd^ level recursion of Leiden genetic communities in Ireland and Britain. We then ran the IBDNe program [(23)](https://www.zotero.org/google-docs/?bCYnAH) on this dataset with *nboots*=100 to generate confidence intervals of the Ne estimates.

### Migration Surface Estimation

We first limited the IBD sharing summary dataset to pairs of individuals both of whom had associated geographic data. It was further subset by IBD length to determine changes in migration surfaces over time: [1,5cM) for migration rates from approximately 100 generations ago and ≥5cM for migration rates from approximately 15 generations ago. A square matrix that captured the number of IBD segments shared between pairs of individuals was created. With the IBD sharing matrix, a file with the geographic coordinates of every individual and shape file with the coordinates of the outline of the Irish and British borders as input, we ran Migration and Population Surface (MAPS) [(24)](https://www.zotero.org/google-docs/?L11rX5) to estimate time-resolved dispersal rates and population densities. We set nDemes = 300, numMCMCIter = 1x10^6^, numBurnIter = 5x10^5^ and numThinIter = 100 over 5 parallel chains. If the log-likelihood values from all the chains didn’t converge to similar values, we used results from the chain with the highest log-likelihood value to initialise the next round of estimations with the same parameters until the log-likelihood values converged. The figures were generated using the *plotmaps* package [(24)](https://www.zotero.org/google-docs/?E4sx0E).

### Estimating Timeline of European Ancestry in Ireland and Britain

After combining the WTCCC2-MS dataset with the Irish-British dataset, we detected IBD segments and sorted the IBD segments into length bins of [1,3cM), [3,5cM) and ≥5cM. With the regional European reference ancestry labels [(1)](https://www.zotero.org/google-docs/?zCi22f) generated using fineSTRUCTURE, we summarised the extent of IBD shared between each European reference ancestry population and the 3rd level recursion of genetic communities within Ireland and Britain within the IBD length bins. We then generated PCA biplots [(25)](https://www.zotero.org/google-docs/?ad389c) from this data to detect the temporal contributions of ancestry from the reference European populations. We also generated heatmaps after scaling the average total length and the number of IBD segments shared between pairs of populations.

### Surname Analysis

Through the Irish DNA Atlas study, we had access to the surnames of the eight great-grandparents of every participant. These surnames were classified into 10 groups based on their origin (Supplemental Table 8) by Irish genealogists (S.O’R and M.M). Those whose surnames were unknown/ambiguous were assigned into the “Unknown” category. We first assigned the genetic community label to each great-grandparent and then created a two-way frequency table of the number of surnames by their origin and the genetic group to which they belonged. Using this table as input, we used the *assoc* function in the *vcd* package [(26,27)](https://www.zotero.org/google-docs/?FAaJKE) (ver. 1.4-11) to identify which surnames are enriched or depleted within each genetic community that uses residuals from a Chi-square test to infer the results.

## Supplemental Note 1

The results from *pvclust* show that the Irish communities resolved along geographic boundaries. The branch consisting of South Munster and North Kerry was similar to the communities from Connacht, North Leinster and North-West Ireland. Interestingly, North Munster grouped together with communities from Wexford, West Leinster, and Dublin and not with other Munster communities. Within the UK sub-branch, we observed the Northern Irish communities group together with North England and North-East Scotland. The Welsh and Cornish communities are grouped in their own sub-branches respectively. England formed its own sub-branch, where the English community split into sub-communities with individuals with ancestry from East Anglia, the Midlands and South England grouped separately, and branched with individuals with ancestry from Border Wales and Devon. The certainty of these broad Irish and British branches of the tree were confirmed using 10,000 bootstrap resampling with the *pvclust* implementation of *hclust*.

This reproducibility analysis of genetic community detection showed that reproducibility decreases with every recursion of the Leiden community detection algorithm. The median reproducibility of the first recursion was 99.95%. For the second recursion, the median reproducibility was 87.52% and 74.47% for Irish and British communities respectively. However, reproducibility was drastically reduced for the third recursion: it ranged between 15.82 to 38.41% for the Irish communities and 25.09 to 78.01% for the British communities (Supplemental Table 1). We further tested robustness through F_ST_ distances between our British and Irish communities. The analysis demonstrated subtle genetic differences between the communities with the greatest differentiation observed between the Orcadian and non-Orcadian communities (mean F_ST_ of 0.00186 between the Orkney community and the other Irish and British communities), in agreement with previous reports[(1,4,18,28)](https://www.zotero.org/google-docs/?Ry4dNQ). Interestingly, the North Wales (mean F_ST_ = 0.00175) and Isle of Man (mean F_ST_ = 0.00165) communities also have relatively higher levels of separation compared to the other Irish and British genetic communities (Supplemental Table 2). Within Ireland, the North Kerry, South Munster, and Wexford communities differed the most genetically from the other Irish communities. Genetic communities from South Leinster were similar to communities from Connacht-Leinster. The English and the Cornish communities showed more subtle genetic differentiation compared to the Orcadian, Welsh and Cornish communities. Total-variation distance (TVD) measures confirm that the communities detected by the Leiden algorithm are indeed robust — the p-values are significantly lesser than 0.01 (Supplemental Table 3).

## Supplemental Note 2

Demographic histories of the regional genetic communities were inferred by analysing IBD and ROH segments in length bins. We estimated the expected segment age for each length bin using equation s19 from reference [(20)](https://www.zotero.org/google-docs/?Tg43Cu)(reproduced here), assuming a large effective population size[(20)](https://www.zotero.org/google-docs/?FtpAUl).


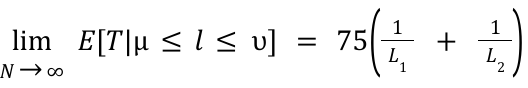


Where $T$ is coalescence time (generations), $l$ is segment length (base pairs), $\mu$ and $\upsilon$ are the upper and lower segment length bounds of the bin (base pairs) and $L_{1}$ and $L_{2}$ are the upper and lower bounds rescaled to centiMorgan.

We chose to split ROH and IBD segments into the corresponding bins (a) 1 to 3cM [1,3cM), (b) 3 to 5cM [3,5cM), and (c) greater than or equal to 5cM (≥5cM). Using the methodology above[(20)](https://www.zotero.org/google-docs/?7CFKQ1), these length bins correspond to 100 generations ago, 40 generations ago and 15 generations ago respectively. An important caveat is that the estimates have very wide distributions, as well as the aforementioned assumption of population size.

# REFERENCES

[1. Gilbert E, O’Reilly S, Merrigan M, McGettigan D, Molloy AM, Brody LC, et al. The Irish DNA Atlas: Revealing Fine-Scale Population Structure and History within Ireland. Sci Rep. 2017 Dec 8;7(1):17199.](https://www.zotero.org/google-docs/?2uw1WP)

[2. Desch KC, Ozel AB, Siemieniak D, Kalish Y, Shavit JA, Thornburg CD, et al. Linkage analysis identifies a locus for plasma von Willebrand factor undetected by genome-wide association. Proc Natl Acad Sci U S A. 2013 Jan 8;110(2):588–93.](https://www.zotero.org/google-docs/?2uw1WP)

[3. van Rheenen W, Shatunov A, Dekker AM, McLaughlin RL, Diekstra FP, Pulit SL, et al. Genome-wide association analyses identify new risk variants and the genetic architecture of amyotrophic lateral sclerosis. Nat Genet. 2016 Sep;48(9):1043–8.](https://www.zotero.org/google-docs/?2uw1WP)

[4. Leslie S, Winney B, Hellenthal G, Davison D, Boumertit A, Day T, et al. The fine scale genetic structure of the British population. Nature. 2015 Mar 19;519(7543):309–14.](https://www.zotero.org/google-docs/?2uw1WP)

[5. Bycroft C, Freeman C, Petkova D, Band G, Elliott LT, Sharp K, et al. The UK Biobank resource with deep phenotyping and genomic data. Nature. 2018 Oct;562(7726):203–9.](https://www.zotero.org/google-docs/?2uw1WP)

[6. Ester M, Kriegel HP, Sander J, Xu X. A density-based algorithm for discovering clusters in large spatial databases with noise. In: Proceedings of the Second International Conference on Knowledge Discovery and Data Mining. Portland, Oregon: AAAI Press; 1996. p. 226–31. (KDD’96).](https://www.zotero.org/google-docs/?2uw1WP)

[7. Purcell S, Neale B, Todd-Brown K, Thomas L, Ferreira MAR, Bender D, et al. PLINK: A Tool Set for Whole-Genome Association and Population-Based Linkage Analyses. Am J Hum Genet. 2007 Sep;81(3):559–75.](https://www.zotero.org/google-docs/?2uw1WP)

[8. Anderson CA, Pettersson FH, Clarke GM, Cardon LR, Morris AP, Zondervan KT. Data quality control in genetic case-control association studies. Nat Protoc. 2010 Sep;5(9):1564–73.](https://www.zotero.org/google-docs/?2uw1WP)

[9. Gilbert E, Zurel H, MacMillan ME, Demiriz S, Mirhendi S, Merrigan M, et al. The Newfoundland and Labrador mosaic founder population descends from an Irish and British diaspora from 300 years ago. Commun Biol. 2023 Apr 28;6(1):1–12.](https://www.zotero.org/google-docs/?2uw1WP)

[10. Delaneau O, Zagury JF, Robinson MR, Marchini JL, Dermitzakis ET. Accurate, scalable and integrative haplotype estimation. Nat Commun. 2019 Nov 28;10(1):5436.](https://www.zotero.org/google-docs/?2uw1WP)

[11. Browning BL, Browning SR. Improving the Accuracy and Efficiency of Identity-by-Descent Detection in Population Data. Genetics. 2013 Jun;194(2):459–71.](https://www.zotero.org/google-docs/?2uw1WP)

[12. Revealing the recent demographic history of Europe via haplotype sharing in the UK Biobank | PNAS [Internet]. [cited 2022 Dec 19]. Available from: https://www.pnas.org/doi/abs/10.1073/pnas.2119281119?url_ver=Z39.88-2003&rfr_id=ori%3Arid%3Acrossref.org&rfr_dat=cr_pub++0pubmed](https://www.zotero.org/google-docs/?2uw1WP)

[13. Csardi G, Nepusz T. The igraph software package for complex network research.](https://www.zotero.org/google-docs/?2uw1WP)

[14. Traag VA, Waltman L, van Eck NJ. From Louvain to Leiden: guaranteeing well-connected communities. Sci Rep. 2019 Mar 26;9(1):5233.](https://www.zotero.org/google-docs/?2uw1WP)

[15. Kharchenko P, Petukhov V, Biederstedt E. leidenAlg: Implements the Leiden Algorithm via an R Interface. R package version 1.0.5. [Internet]. 2021. Available from: https://github.com/kharchenkolab/leidenAlg](https://www.zotero.org/google-docs/?2uw1WP)

[16. Yang Y, Durbin R, Iversen AKN, Lawson DJ. Sparse haplotype-based fine-scale local ancestry inference at scale reveals recent selection on immune responses [Internet]. medRxiv; 2024 [cited 2024 Apr 4]. p. 2024.03.13.24304206. Available from: https://www.medrxiv.org/content/10.1101/2024.03.13.24304206v1](https://www.zotero.org/google-docs/?2uw1WP)

[17. Maier R, Isildak U. ADMIXTOOLS 2, [Internet]. Available from: https://github.com/uqrmaie1/admixtools](https://www.zotero.org/google-docs/?2uw1WP)

[18. Byrne RP, Martiniano R, Cassidy LM, Carrigan M, Hellenthal G, Hardiman O, et al. Insular Celtic population structure and genomic footprints of migration. PLOS Genet. 2018 Jan 25;14(1):e1007152.](https://www.zotero.org/google-docs/?2uw1WP)

[19. Meyer  [aut D, cre, Dimitriadou E, Hornik K, Weingessel A, Leisch F, et al. e1071: Misc Functions of the Department of Statistics, Probability Theory Group (Formerly: E1071), TU Wien [Internet]. 2023 [cited 2023 Aug 29]. Available from: https://cran.r-project.org/web/packages/e1071/index.html](https://www.zotero.org/google-docs/?2uw1WP)

[20. Byrne RP, van Rheenen W, van den Berg LH, Veldink JH, McLaughlin RL. Dutch population structure across space, time and GWAS design. Nat Commun. 2020 Sep 11;11(1):4556.](https://www.zotero.org/google-docs/?2uw1WP)

[21. Nei M. Definition and Estimation of Fixation Indices. Evolution. 1986;40(3):643–5.](https://www.zotero.org/google-docs/?2uw1WP)

[22. McQuillan R, Eklund N, Pirastu N, Kuningas M, McEvoy BP, Esko T, et al. Evidence of Inbreeding Depression on Human Height. PLoS Genet. 2012 Jul 19;8(7):e1002655.](https://www.zotero.org/google-docs/?2uw1WP)

[23. Browning SR, Browning BL. Accurate Non-parametric Estimation of Recent Effective Population Size from Segments of Identity by Descent. Am J Hum Genet. 2015 Sep 3;97(3):404–18.](https://www.zotero.org/google-docs/?2uw1WP)

[24. Al-Asadi H, Petkova D, Stephens M, Novembre J. Estimating recent migration and population-size surfaces. PLOS Genet. 2019 Jan 14;15(1):e1007908.](https://www.zotero.org/google-docs/?2uw1WP)

[25. Kassambara A, Mundt F. factoextra: Extract and Visualize the Results of Multivariate Data Analyses [Internet]. 2020 [cited 2023 Aug 29]. Available from: https://cran.r-project.org/web/packages/factoextra/index.html](https://www.zotero.org/google-docs/?2uw1WP)

[26. Zeileis A, Meyer D, Hornik K. Residual-Based Shadings for Visualizing (Conditional) Independence. J Comput Graph Stat. 2007 Sep 1;16(3):507–25.](https://www.zotero.org/google-docs/?2uw1WP)

[27. Meyer  [aut D, cre, Zeileis A, Hornik K, Gerber F, Friendly M. vcd: Visualizing Categorical Data [Internet]. 2023 [cited 2023 Aug 4]. Available from: https://cran.r-project.org/web/packages/vcd/index.html](https://www.zotero.org/google-docs/?2uw1WP)

[28. O’Dushlaine CT, Morris D, Moskvina V, Kirov G, Consortium IS, Gill M, et al. Population structure and genome-wide patterns of variation in Ireland and Britain. Eur J Hum Genet. 2010 Nov;18(11):1248–54.](https://www.zotero.org/google-docs/?2uw1WP)
