# Supplementary material for: A genetic perspective on the recent demographic history of Ireland and Britain
Source: Eur J Hum Genet. 2025 Feb 5;33(4):538–45. doi: 10.1038/s41431-025-01794-0 (PMC11986122; doi:10.1038/s41431-025-01794-0)
Supplement: Supplementary file 2 — Supplemetal Figures [file 41431_2025_1794_MOESM2_ESM.pptx]

## Slide 1
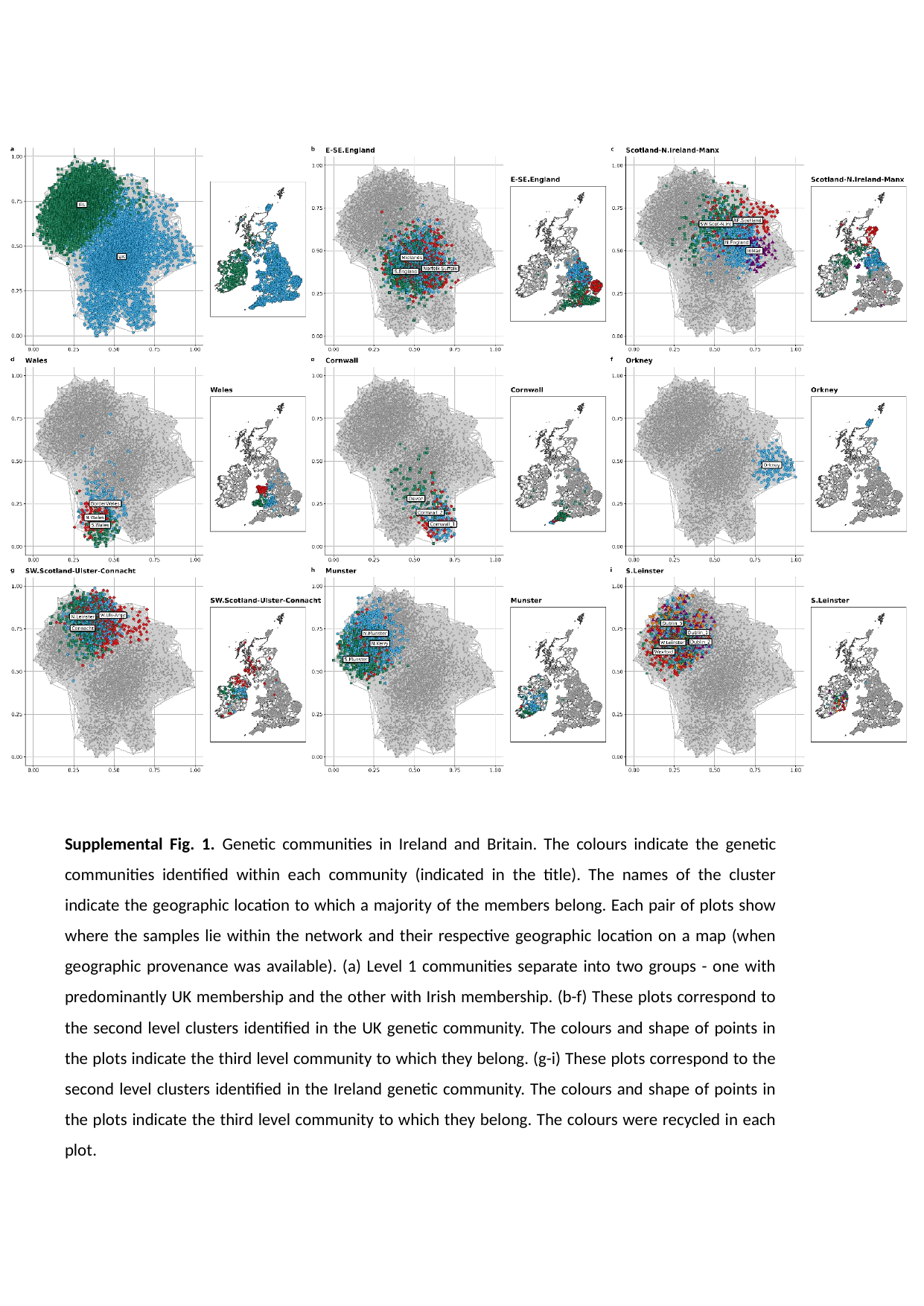

Supplemental Fig. 1. Genetic communities in Ireland and Britain. The colours indicate the genetic communities identified within each community (indicated in the title). The names of the cluster indicate the geographic location to which a majority of the members belong. Each pair of plots show where the samples lie within the network and their respective geographic location on a map (when geographic provenance was available). (a) Level 1 communities separate into two groups - one with predominantly UK membership and the other with Irish membership. (b-f) These plots correspond to the second level clusters identified in the UK genetic community. The colours and shape of points in the plots indicate the third level community to which they belong. (g-i) These plots correspond to the second level clusters identified in the Ireland genetic community. The colours and shape of points in the plots indicate the third level community to which they belong. The colours were recycled in each plot.

## Slide 2
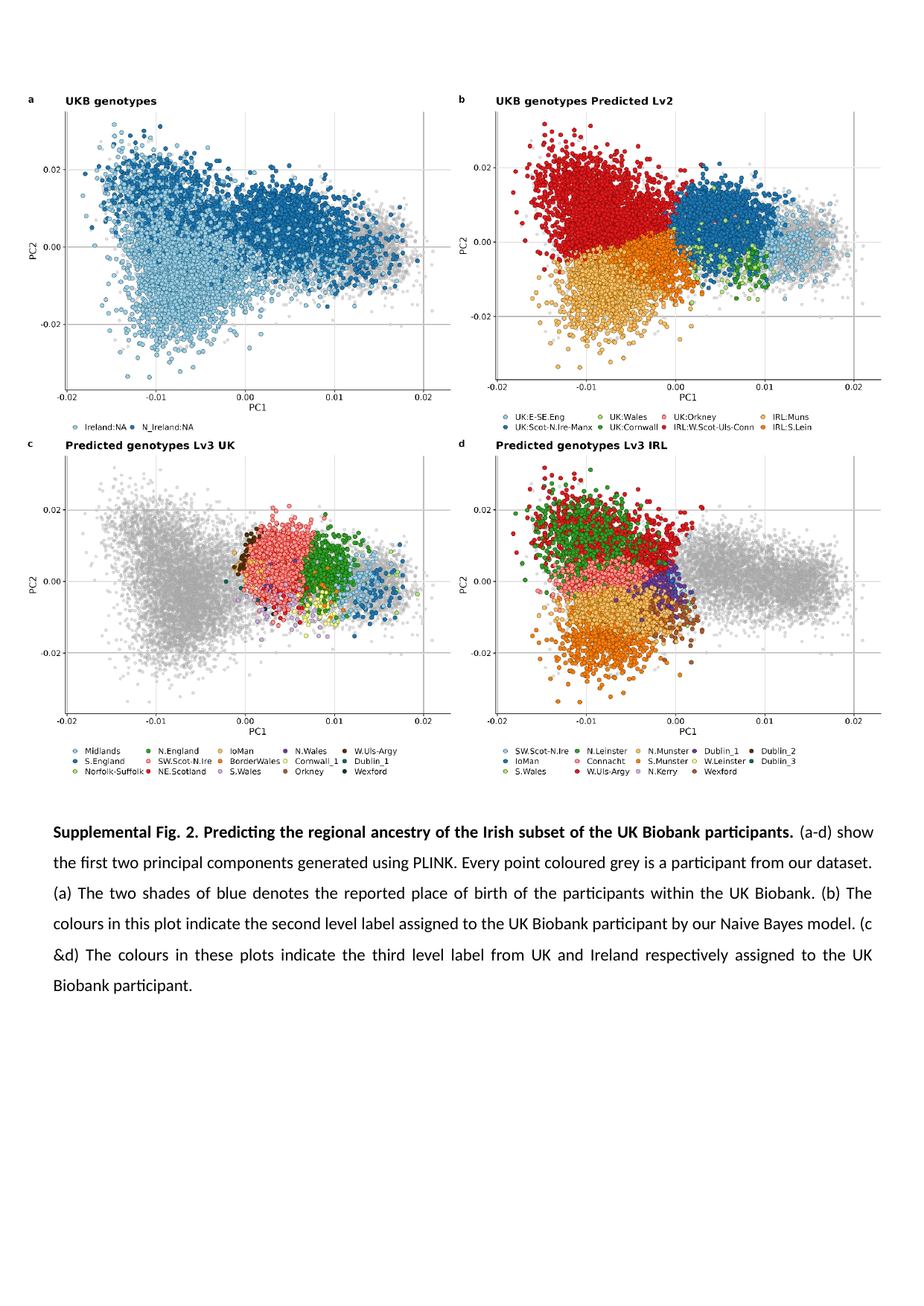

Supplemental Fig. 2. Predicting the regional ancestry of the Irish subset of the UK Biobank participants. (a-d) show the first two principal components generated using PLINK. Every point coloured grey is a participant from our dataset. (a) The two shades of blue denotes the reported place of birth of the participants within the UK Biobank. (b) The colours in this plot indicate the second level label assigned to the UK Biobank participant by our Naive Bayes model. (c &d) The colours in these plots indicate the third level label from UK and Ireland respectively assigned to the UK Biobank participant.

## Slide 3
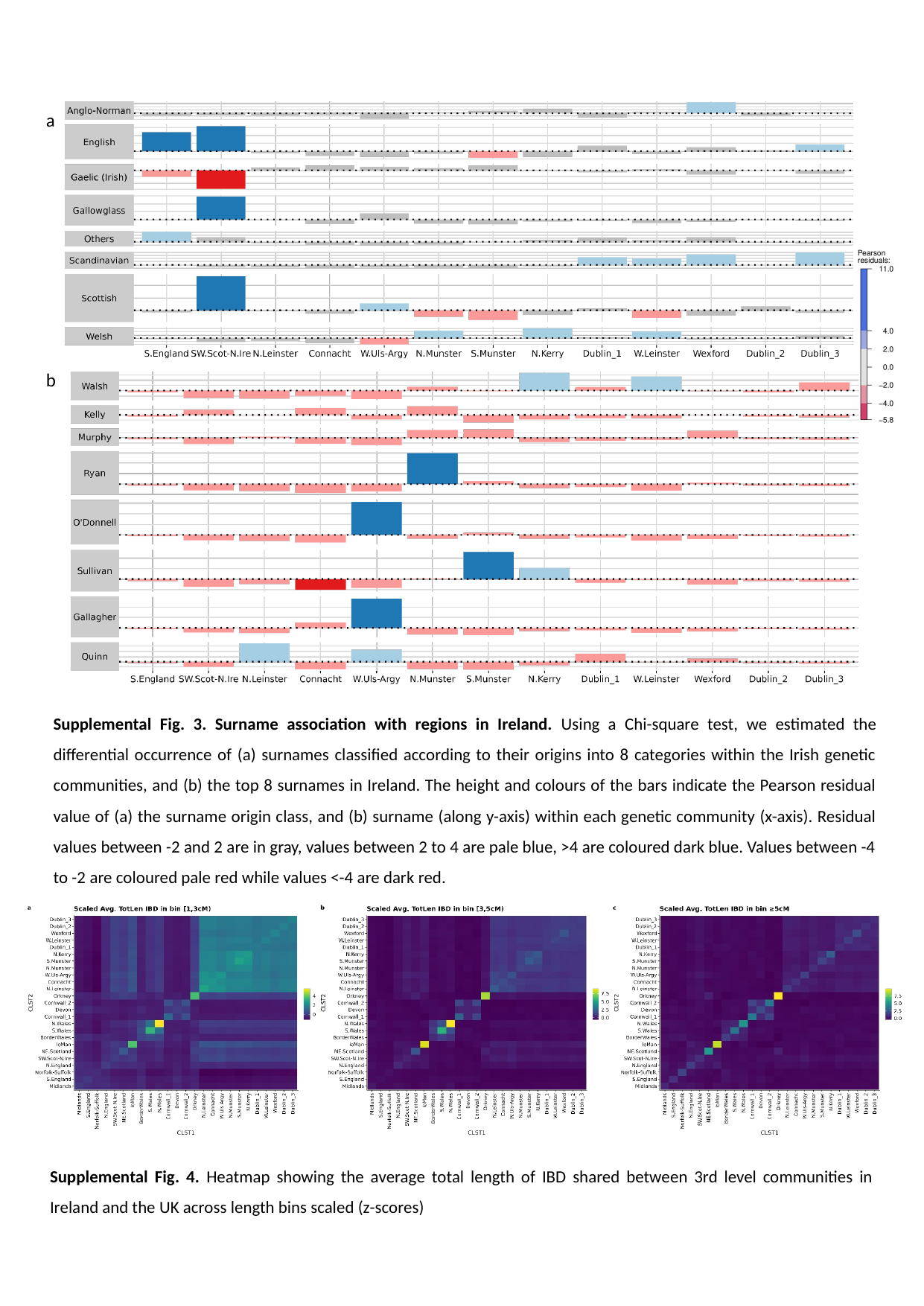

a
b
Supplemental Fig. 3. Surname association with regions in Ireland. Using a Chi-square test, we estimated the differential occurrence of (a) surnames classified according to their origins into 8 categories within the Irish genetic communities, and (b) the top 8 surnames in Ireland. The height and colours of the bars indicate the Pearson residual value of (a) the surname origin class, and (b) surname (along y-axis) within each genetic community (x-axis). Residual values between -2 and 2 are in gray, values between 2 to 4 are pale blue, >4 are coloured dark blue. Values between -4 to -2 are coloured pale red while values <-4 are dark red.
Supplemental Fig. 4. Heatmap showing the average total length of IBD shared between 3rd level communities in Ireland and the UK across length bins scaled (z-scores)

## Slide 4
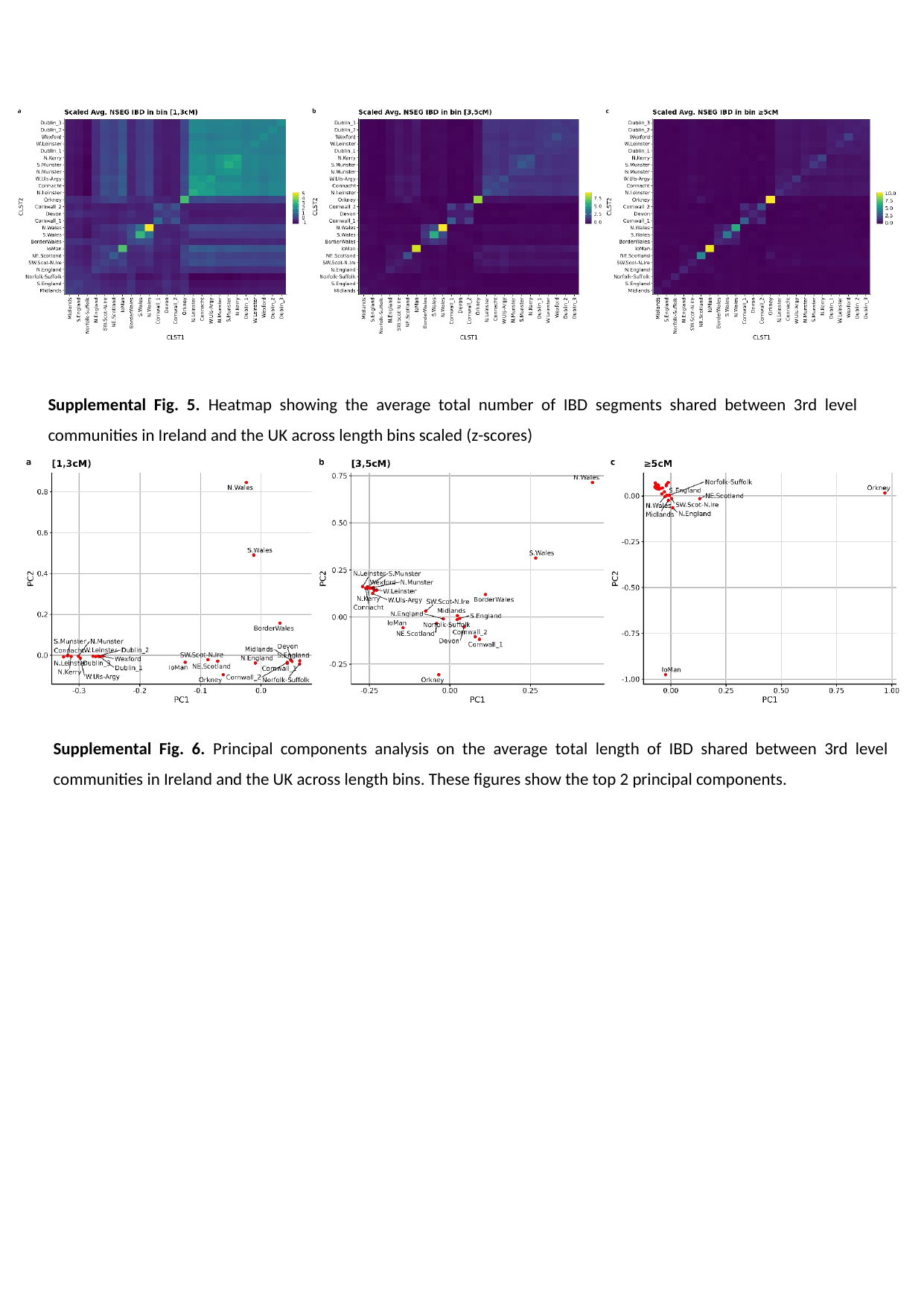

Supplemental Fig. 5. Heatmap showing the average total number of IBD segments shared between 3rd level communities in Ireland and the UK across length bins scaled (z-scores)
Supplemental Fig. 6. Principal components analysis on the average total length of IBD shared between 3rd level communities in Ireland and the UK across length bins. These figures show the top 2 principal components.

## Slide 5
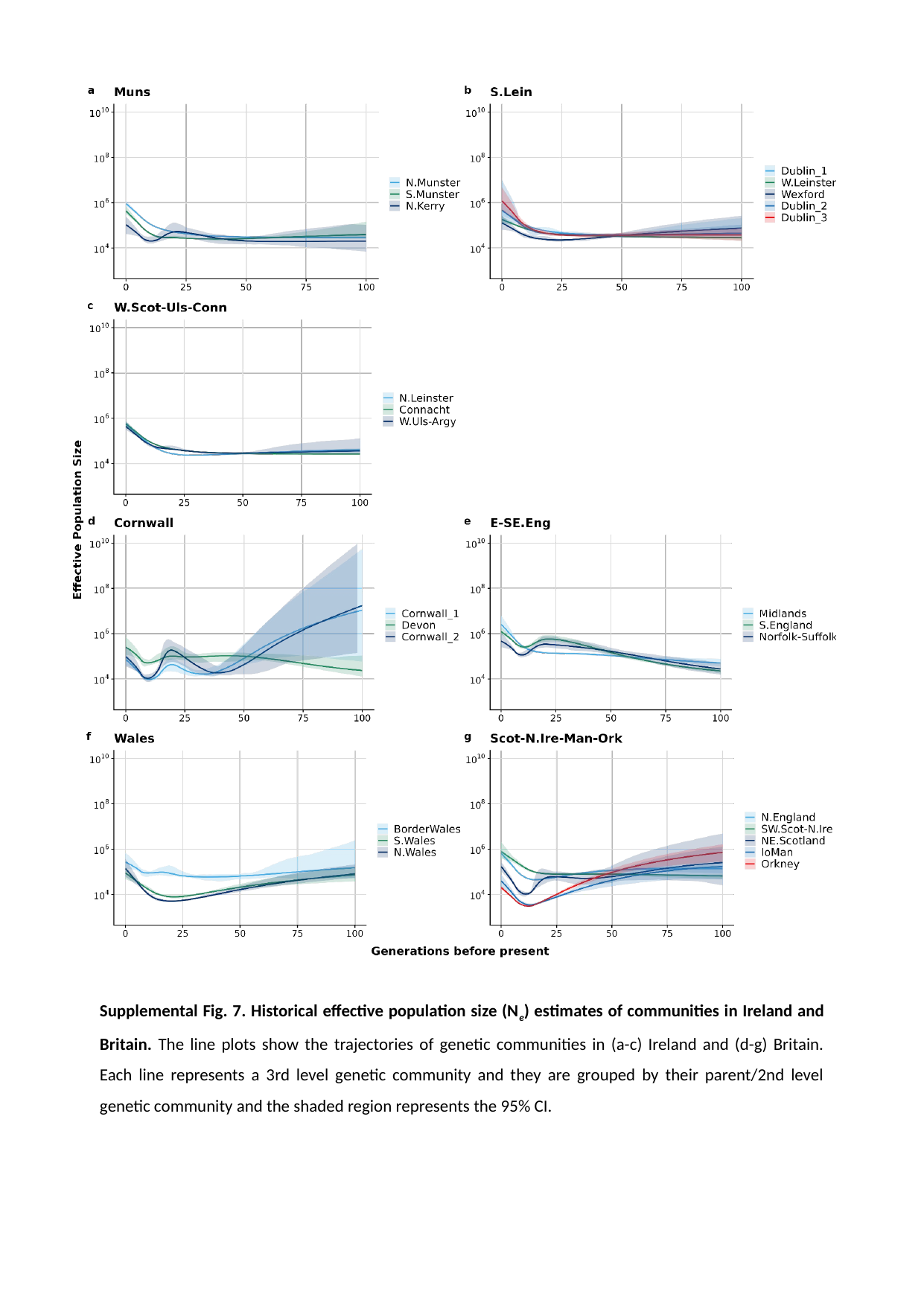

Supplemental Fig. 7. Historical effective population size (Ne) estimates of communities in Ireland and Britain. The line plots show the trajectories of genetic communities in (a-c) Ireland and (d-g) Britain. Each line represents a 3rd level genetic community and they are grouped by their parent/2nd level genetic community and the shaded region represents the 95% CI.

## Slide 6
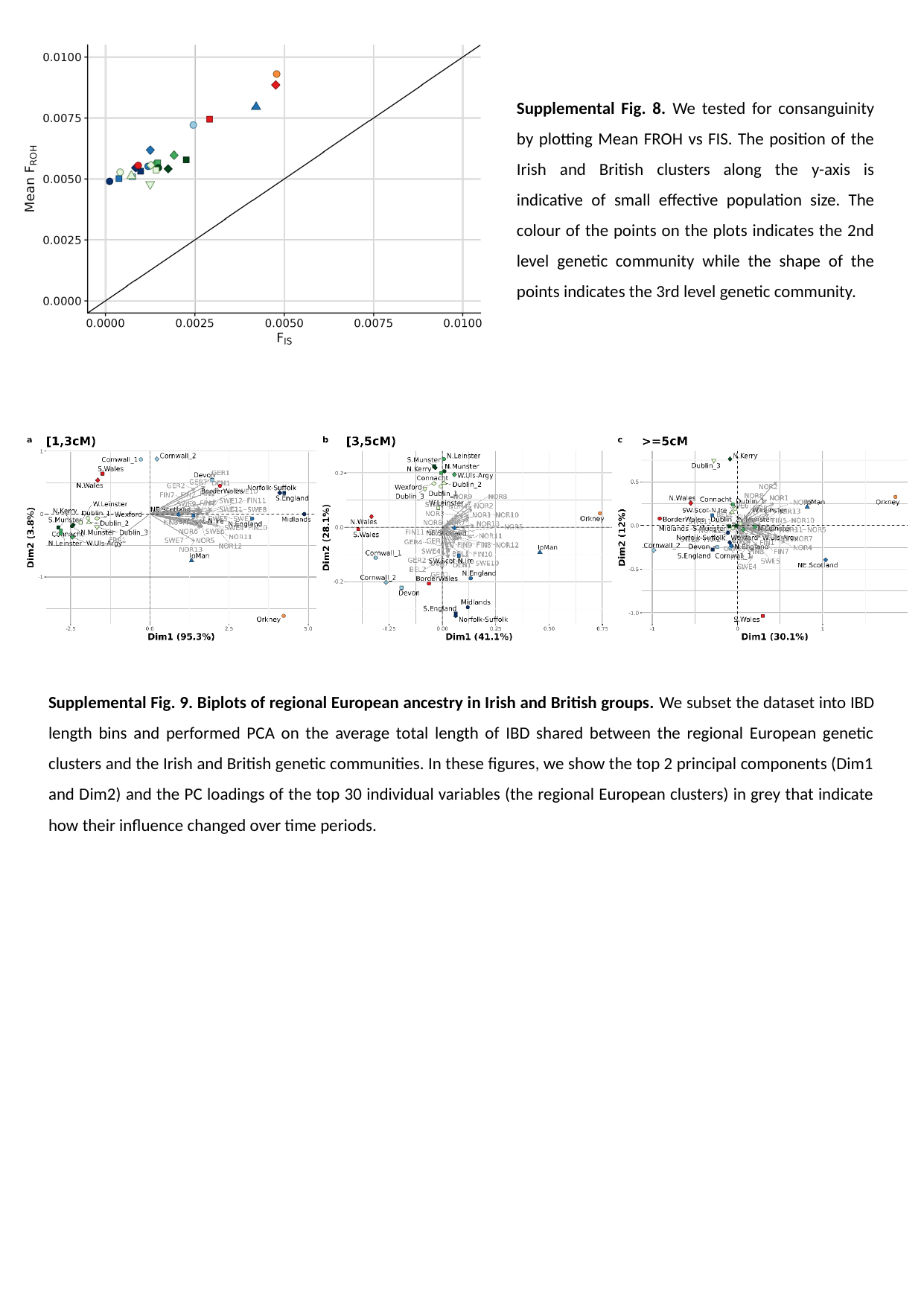

Supplemental Fig. 8. We tested for consanguinity by plotting Mean FROH vs FIS. The position of the Irish and British clusters along the y-axis is indicative of small effective population size. The colour of the points on the plots indicates the 2nd level genetic community while the shape of the points indicates the 3rd level genetic community.
Supplemental Fig. 9. Biplots of regional European ancestry in Irish and British groups. We subset the dataset into IBD length bins and performed PCA on the average total length of IBD shared between the regional European genetic clusters and the Irish and British genetic communities. In these figures, we show the top 2 principal components (Dim1 and Dim2) and the PC loadings of the top 30 individual variables (the regional European clusters) in grey that indicate how their influence changed over time periods.

## Slide 7
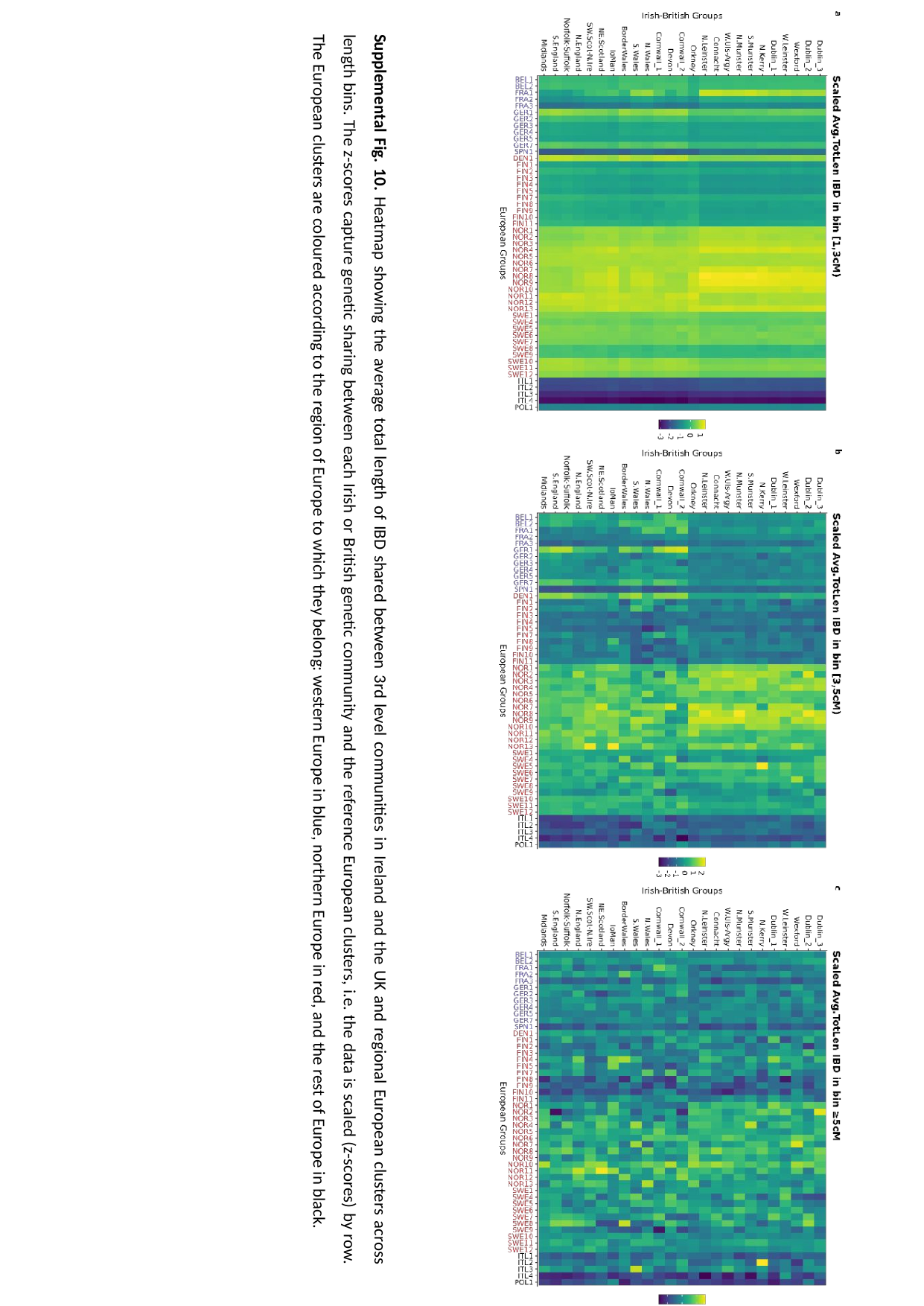

Supplemental Fig. 10. Heatmap showing the average total length of IBD shared between 3rd level communities in Ireland and the UK and regional European clusters across length bins. The z-scores capture genetic sharing between each Irish or British genetic community and the reference European clusters, i.e. the data is scaled (z-scores) by row. The European clusters are coloured according to the region of Europe to which they belong: western Europe in blue, northern Europe in red, and the rest of Europe in black.

## Slide 8
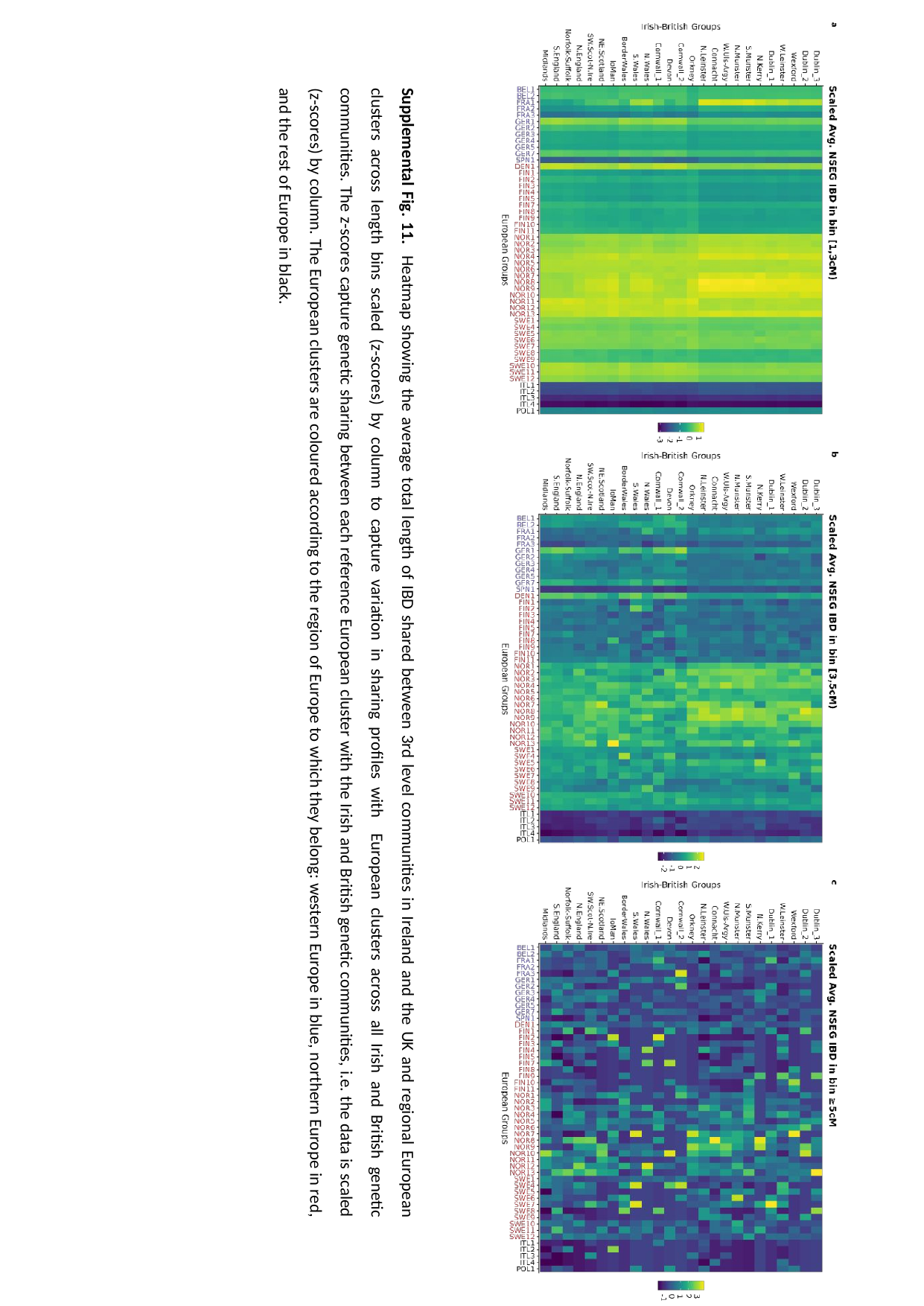

Supplemental Fig. 11. Heatmap showing the average total length of IBD shared between 3rd level communities in Ireland and the UK and regional European clusters across length bins scaled (z-scores) by column to capture variation in sharing profiles with European clusters across all Irish and British genetic communities. The z-scores capture genetic sharing between each reference European cluster with the Irish and British genetic communities, i.e. the data is scaled (z-scores) by column. The European clusters are coloured according to the region of Europe to which they belong: western Europe in blue, northern Europe in red, and the rest of Europe in black.

## Slide 9
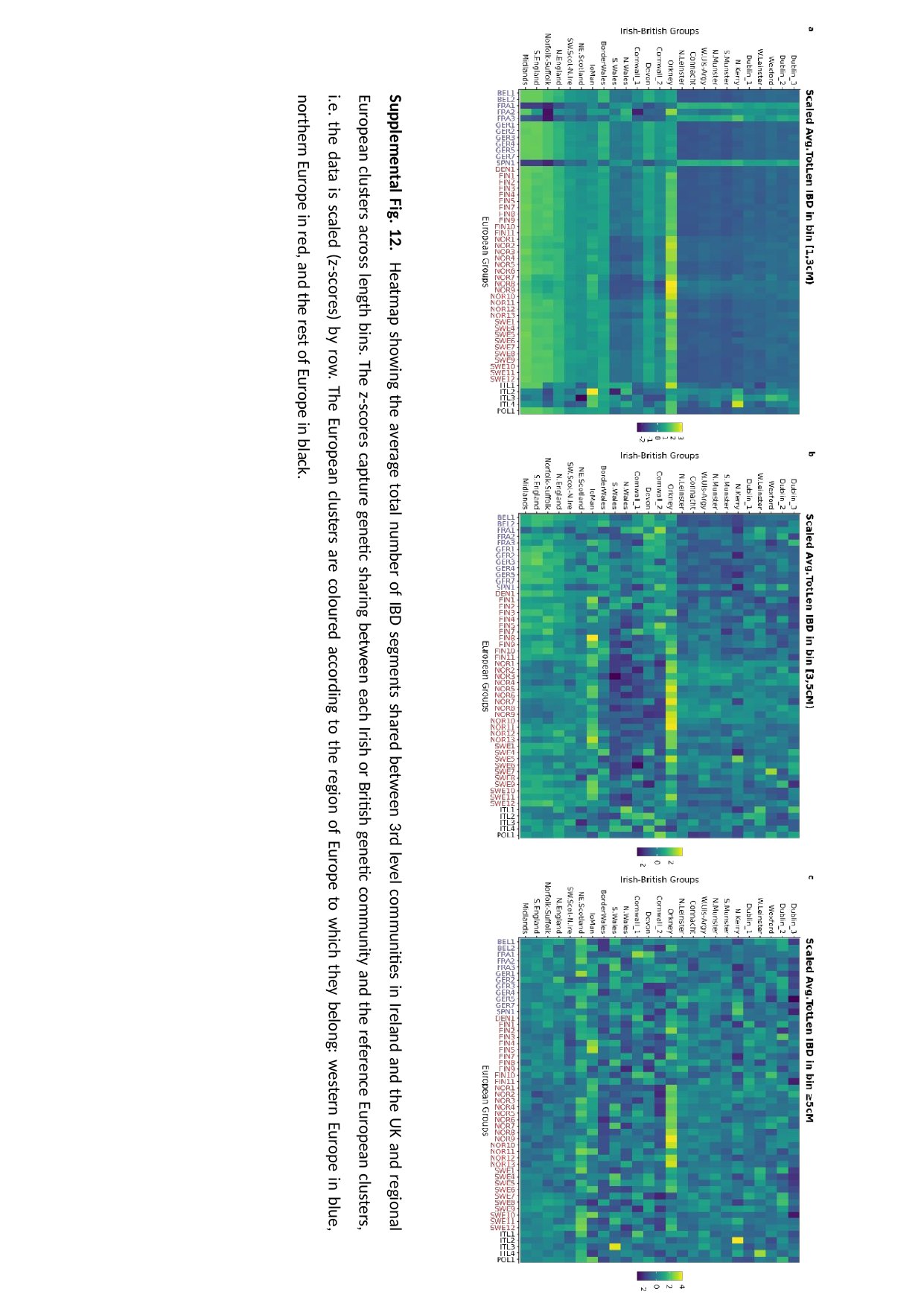

Supplemental Fig. 12. Heatmap showing the average total number of IBD segments shared between 3rd level communities in Ireland and the UK and regional European clusters across length bins. The z-scores capture genetic sharing between each Irish or British genetic community and the reference European clusters, i.e. the data is scaled (z-scores) by row. The European clusters are coloured according to the region of Europe to which they belong: western Europe in blue, northern Europe in red, and the rest of Europe in black.

## Slide 10
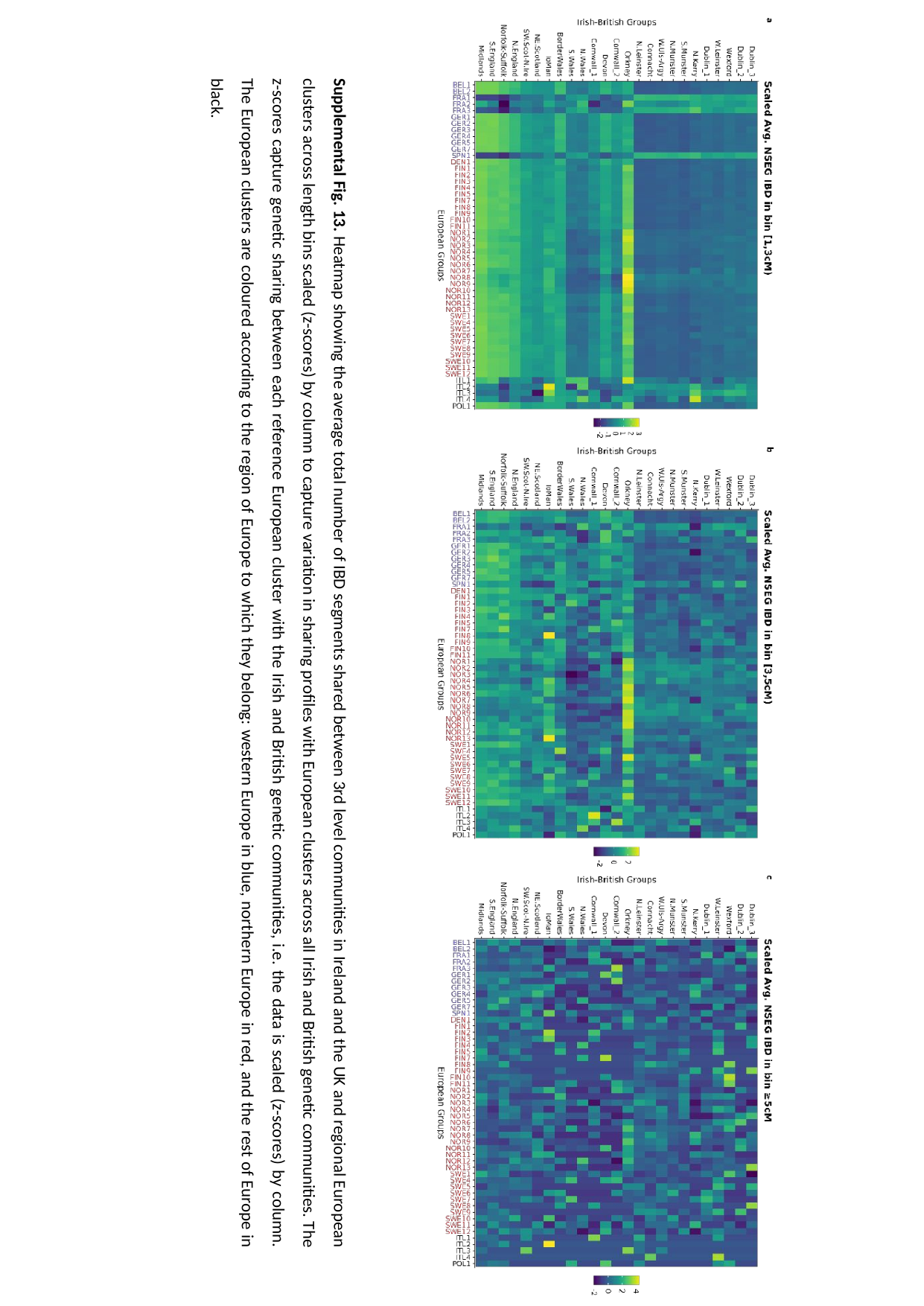

Supplemental Fig. 13. Heatmap showing the average total number of IBD segments shared between 3rd level communities in Ireland and the UK and regional European clusters across length bins scaled (z-scores) by column to capture variation in sharing profiles with European clusters across all Irish and British genetic communities. The z-scores capture genetic sharing between each reference European cluster with the Irish and British genetic communities, i.e. the data is scaled (z-scores) by column. The European clusters are coloured according to the region of Europe to which they belong: western Europe in blue, northern Europe in red, and the rest of Europe in black.
